# Real-time detection and monitoring of bacteria in diabetic wounds using bacterial fluorescence

**DOI:** 10.3389/fmicb.2026.1867420

**Published:** 2026-07-03

**Authors:** Xiaofen Sun, Ao Du, Meili Dong, Yuanzhi Zhang, Yang Zhang, Yao Huang, Xiang Huang, Mingwei Cheng, Yikun Wang, Yong Liu, Jingshu Ni

**Affiliations:** 1 Anhui Institute of Optics and Fine Mechanics, Hefei Institutes of Physical Science, Chinese Academy of Sciences, Hefei, Anhui, China; 2 University of Science and Technology of China, Hefei, Anhui, China; 3 Wanjiang Emerging Industry Technology Development Center, Tongling, Anhui, China; 4 Division of Life Sciences and Medicine, Department of Anesthesiology, First Affiliated Hospital of USTC, University of Science and Technology of China, Hefei, Anhui, China; 5 Department of Endocrinology, The First Affiliated Hospital of Anhui Medical University, Hefei, Anhui, China

**Keywords:** antimicrobial treatment, bacterial burden, bacterial fluorescence, diabetic wound, wound healing

## Abstract

**Background:**

Real-time monitoring of bacterial dynamics is essential for preventing the progression of diabetic foot infections (DFIs). This study characterizes bacterial fluorescence in infected diabetic wounds and evaluates its utility for real-time infection surveillance during antimicrobial treatment.

**Methods:**

A diabetic rat model was established, and full-thickness wounds were inoculated with three representative bacterial species. Antimicrobial interventions were subsequently administered. Bacterial fluorescence, bacterial burden, and wound status were longitudinally assessed.

**Results:**

Inoculation with 10^3^ CFU was sufficient to establish infection, enabling *in vivo* visualization of bacterial fluorescence, which emerged 1–3 days prior to the onset of clinical signs. Fluorescence intensity showed a strong association with both swab- and tissue-derived bacterial burden, and persistent fluorescence was associated with sustained infection and delayed healing. In *Escherichia coli* and *Staphylococcus aureus* infections, both antibiotic therapy and debridement markedly reduced bacterial burden, decreased fluorescence intensity, attenuated inflammation, and improved wound healing with comparable efficacy. In contrast, *Pseudomonas aeruginosa* infections exhibited recurrent fluorescence and bacterial regrowth following antibiotic therapy, accompanied by persistent inflammation and impaired tissue repair, whereas debridement provided superior bacterial control and more favorable healing outcomes.

**Conclusion:**

These findings elucidate the relationship between bacterial fluorescence and infection dynamics and suggest that fluorescence imaging may serve as a real-time, noninvasive tool for detecting and monitoring diabetic wound infections.

## 1 Introduction

Recent estimates indicate that as of 2024, approximately 589 million adults worldwide were living with diabetes, a number projected to surge to 853 million by 2050 (Genitsaridi et al., 2026). Approximately one-third of individuals with diabetes will develop a diabetic foot ulcer (DFU), and nearly half of these ulcers become infected during their course. Once infection occurs, patients experience markedly increased rates of hospitalization, disability, and mortality (Lo et al., 2021; Raspovic and Wukich, 2014). Therefore, early diagnosis and effective infection control are central to the management of diabetic foot infections (DFIs).

Current clinical diagnosis of DFIs relies on clinical assessment, laboratory tests, and imaging. Clinical assessment primarily relies on the visual and tactile evaluation of clinical signs and symptoms (CSS), including local swelling, erythema, tenderness, warmth, and purulent exudate (Prompers et al., 2007). However, in patients with diabetes, these CSS are often subtle or absent due to neuropathy, peripheral vascular disease, and immune dysfunction. Laboratory tests, such as blood-based diagnostics, swab cultures, and tissue biopsies, are routinely employed to detect causative pathogens and determine their antimicrobial susceptibility (Pereira et al., 2017). Yet, the 2–5 day turnaround time for these tests limits their utility for real-time clinical decision-making. Imaging modalities, while useful for detecting deep infections but typically identify only advanced disease (Dinh et al., 2008). Collectively, conventional approaches are often time-consuming, costly, invasive, and dependent on advanced laboratory infrastructure, thereby significantly limiting the feasibility of real-time or repeated examinations. Consequently, diagnostic delays and prolonged empirical antimicrobial therapy are common in DFIs, and are frequently associated with chronic infection, multidrug resistance, osteomyelitis, and amputation (Lin et al., 2021; Ozer, 2025). Therefore, rapid, non-invasive, point-of-care technologies for real-time infection monitoring are urgently needed.

Fluorescence imaging has emerged as a rapid, non-invasive technique for detecting infected tissue in skin and dental lesions (Seo et al., 2009; van der Veen et al., 2006). More recently, it has been increasingly explored for applications in wound assessment. Upon excitation at specific wavelengths, tissues emit green fluorescence, porphyrin-producing bacteria exhibit red fluorescence, and *Pseudomonas aeruginosa* produces distinctive cyan fluorescence via pyoverdine, enabling discrimination of bacterial fluorescence from background tissue (Rennie et al., 2019). Preclinical studies in nude mice inoculated with 10^7^–10^9^ CFU of *Staphylococcus aureus* or *P. aeruginosa* have demonstrated detectable fluorescence signals (Lopez et al., 2021; Wu et al., 2014). Clinical investigations have shown improved detection of moderate-to-heavy bacterial burden (10^4^–10^9^ CFU/g) using fluorescence imaging, thus recommending fluorescence imaging as an adjunct to standard care in DFIs (Ottolino-Perry et al., 2017; Rahma et al., 2022). However, most studies remain limited to cross-sectional assessments of infected tissues and bacterial fluorescence. The dynamic interplay among bacterial burden, fluorescence signals, host immune responses, and wound status remains poorly characterized, restricting the utility of fluorescence imaging for real-time monitoring and therapeutic guidance.

Accordingly, this study employed a diabetic rat wound infection model with two primary objectives. First, we aimed to characterize the fluorescence properties of three common wound-associated pathogens; Second, we sought to evaluate the temporal changes in bacterial fluorescence, bacterial burden, therapeutic response, and wound status, and to elucidate the relationships among them under antimicrobial interventions.

## 2 Materials and methods

### 2.1 Media and bacteria

Mannitol Salt Agar (MSA), Eosin Methylene Blue (EMB) agar, and *Pseudomonas* cephalothin-sodium fusidate-cetrimide (CFC) agar were purchased from Thermo Fisher Scientific (South San Francisco, CA, United States). Three bacterial strains were obtained from the American Type Culture Collection (ATCC, Manassas, VA, United States): *E. coli* (ATCC 25922), *S. aureus* (ATCC 29213), and *P. aeruginosa* (ATCC 27853).

### 2.2 Instrumentation

We adapted our previously developed bacterial fluorescence imaging device to operate effectively within the confined workspace of a biosafety cabinet (Zheng et al., 2023). The imaging system consisted of a high-sensitivity CCD camera (IMX462, Sony, Japan) and two ultraviolet LED excitation sources (center wavelength: 405 nm; full width at half maximum: 15 nm; optical output power: 1.3 W per LED) (UV 405, Ruichengguangdian, Shenzhen, China). The LEDs were symmetrically mounted at 45° relative to the sample plane with a center-to-center spacing of approximately 8.5 cm to improve illumination uniformity and reduce specular reflection artifacts. A 405 nm band-pass filter (RUICCN-BP405nm, Ruichengguangdian, Shenzhen, China) was integrated into the excitation path for spectral purification. Fluorescence emission was selectively filtered using a custom dual-band emission filter (500–545 nm and 600–650 nm; RUICCN-DB532/630 nm, Ruichengguangdian, Shenzhen, China) prior to acquisition by the CCD sensor, enabling highly sensitive detection of bacterial fluorescence signals. The system was powered by a rechargeable LiPo battery pack (7.4 V, 2,600 mAh) to improve portability. All optical and electronic components were modularly assembled and enclosed in a custom polypropylene housing resistant to disinfection with 75% ethanol. The prototype housing measured approximately 180 mm × 120 mm × 80 mm (length × width × height). The imaging module was bolted to a custom external support frame and positioned approximately 10 cm above the sample platform, providing stable imaging and uniform illumination over an area of approximately 90 mm × 90 mm. The support frame had an overall height of approximately 25 cm and incorporated a 15 cm × 30 cm sample platform at its base. A detailed optical layout of the imaging system is shown in Supplementary Figure 1.

### 2.3 Bacterial preparation

Luria–Bertani (LB) medium consisted of 10 g/L tryptone, 5 g/L yeast extract, and 10 g/L sodium chloride, and was adjusted to pH 7.0. Cryopreserved bacterial strains were streaked onto LB agar plates and incubated to obtain isolated colonies. A single colony was picked and inoculated into 10mL of LB broth and cultured overnight at 37 °C with shaking at 220 rpm. The cultures were centrifuged at 3,000 × g for 10 min at 4°C, and the pellets were washed three times with sterile PBS and resuspended in PBS. Optical density at 600nm (OD_600_) was measured using a spectrophotometer (Infinite 200 Pro MPlex, Tecan, Männedorf, Switzerland), and bacterial concentrations were determined using species-specific OD_600_–CFU calibration curves previously established in our laboratory (Supplementary Figure 2). Standardized suspensions of *E. coli*, *S. aureus*, and *P. aeruginosa* at 1 × 10^4^ CFU/mL were prepared for subsequent experiments.

### 2.4 Kirby–Bauer disc diffusion assay

Antimicrobial susceptibility testing was performed to determine the *in vitro* sensitivity of all bacterial strains used in this study. Briefly, bacterial cell suspensions were adjusted to a 0.5 McFarland standard and swabbed onto Mueller–Hinton agar plates (Sigma–Aldrich, Steinheim, Germany). Imipenem (10 μg) discs were applied according to the Kirby–Bauer disk diffusion method. Plates were incubated at 37 °C for 18 h under aerobic conditions. After incubation, inhibition zone diameters were measured in millimeters and interpreted in accordance with the Clinical and Laboratory Standards Institute (CLSI) guidelines. All strains were classified as susceptible, intermediate, or resistant based on established breakpoint criteria.

### 2.5 *In vitro* observation

Fluorescence of bacterial suspensions and colonies of *E. coli*, *S. aureus*, and *P. aeruginosa* was evaluated *in vitro*. To induce porphyrin production in *E. coli* and *S. aureus*, 5-Aminolevulinic acid (ALA) (Sigma–Aldrich, Steinheim, Germany) was added into LB broth or LB agar at a final concentration of 80 mg/L. *P. aeruginosa* was cultured in LB medium without ALA supplementation. For liquid cultures, 10 μL of standardized bacterial suspension was inoculated into 10 mL of LB broth and incubated with shaking at 37 C° and 220 rpm. For agar cultures, 100 μL of standardized suspensions were evenly spread onto LB agar plates. Both standard and fluorescence images were acquired using our custom-built fluorescence imaging system. Fluorescence imaging was performed in a full darkened environment.

### 2.6 Animal models

All experimental procedures were conducted in strict accordance with the guidelines approved by the Institutional Animal Ethics Subcommittee of the Institute of Health and Medicine, Hefei Comprehensive National Science Center (Approval Number: IHM-AP-2024-011; Approval Date: April 3, 2024). Specific pathogen-free male Sprague–Dawley rats (6–8 weeks old; weighing 250–300 g) were obtained from SiPeiFu Biotechnology (Beijing, China). Animals were housed under controlled environmental conditions (temperature: 20–22C°; relative humidity: 50–70%; 12-h light/dark cycle) with free access to water.

Type 2 diabetes mellitus (T2DM) was induced according to established protocols (Furman, 2015). Briefly, rats were fed a high-fat diet (XTHF60; 60% of total calories from fat, 20% from protein, and 20% from carbohydrates) for 3 weeks to induce insulin resistance, followed by a single intraperitoneal injection of freshly prepared Streptozotocin (STZ, Merck KGaA, Darmstadt, Germany) (40 mg/kg in 50 mM sodium citrate buffer, pH 4.5). The animals were maintained on the high-fat diet throughout the study. Ten days after STZ administration, fasting blood glucose was measured from tail-vein samples using a portable glucometer (Roche Diagnostics, Mannheim, Germany). Rats with fasting blood glucose concentrations exceeding 16.7 mmol/L were considered diabetic.

### 2.7 Wounding, inoculation, and dressing

After confirmation of diabetes, a nonlethal full-thickness excisional wound model was established as previously described (Mendes et al., 2012). Anesthesia was induced with 4% isoflurane and maintained at 2.5% using an RWD-R500 small animal anesthesia system (RWD Life Science, Shenzhen, China). The dorsal area was shaved and depilated using a commercial depilatory cream (Veet, Reckitt Benckiser, France), followed by disinfection with povidone-iodine and 75% alcohol. A 1-cm-diameter full-thickness circular wound was created using a biopsy punch. To minimize wound contraction, a silicone ring (inner diameter: 1.6cm; outer diameter: 2.0cm) was affixed to the wound site using n-butyl-α-cyanoacrylate medical adhesive (Compont Company, Beijing, China) (Wong et al., 2011). Postoperative analgesia was provided via subcutaneous buprenorphine (1 mg/kg).

Immediately after wounding, 100 μL of standardized bacterial suspension (1.0 × 10^3^ CFU in total) was applied to the wound bed in infection groups. The inoculum was delivered using a combined strategy, with half of the suspension injected into the fascial/superficial muscular level and the remaining half applied topically to the wound surface. To ensure sufficient contact of the bacteria with the wound tissue, 15 min was allowed post-inoculation prior to dressing application. Controls received 100 μL of sterile PBS. The wound was then covered with a pre-cut Tegaderm™ transparent film dressing (3M Healthcare, Minnesota, United States) and secured with sterile bandages. All surgeries were performed by the same surgeon to minimize inter-operator variability. Postoperatively, rats were placed on a warming pad (37–42°C) until full recovery and then housed individually.

### 2.8 Experimental design

**FIGURE 1:**
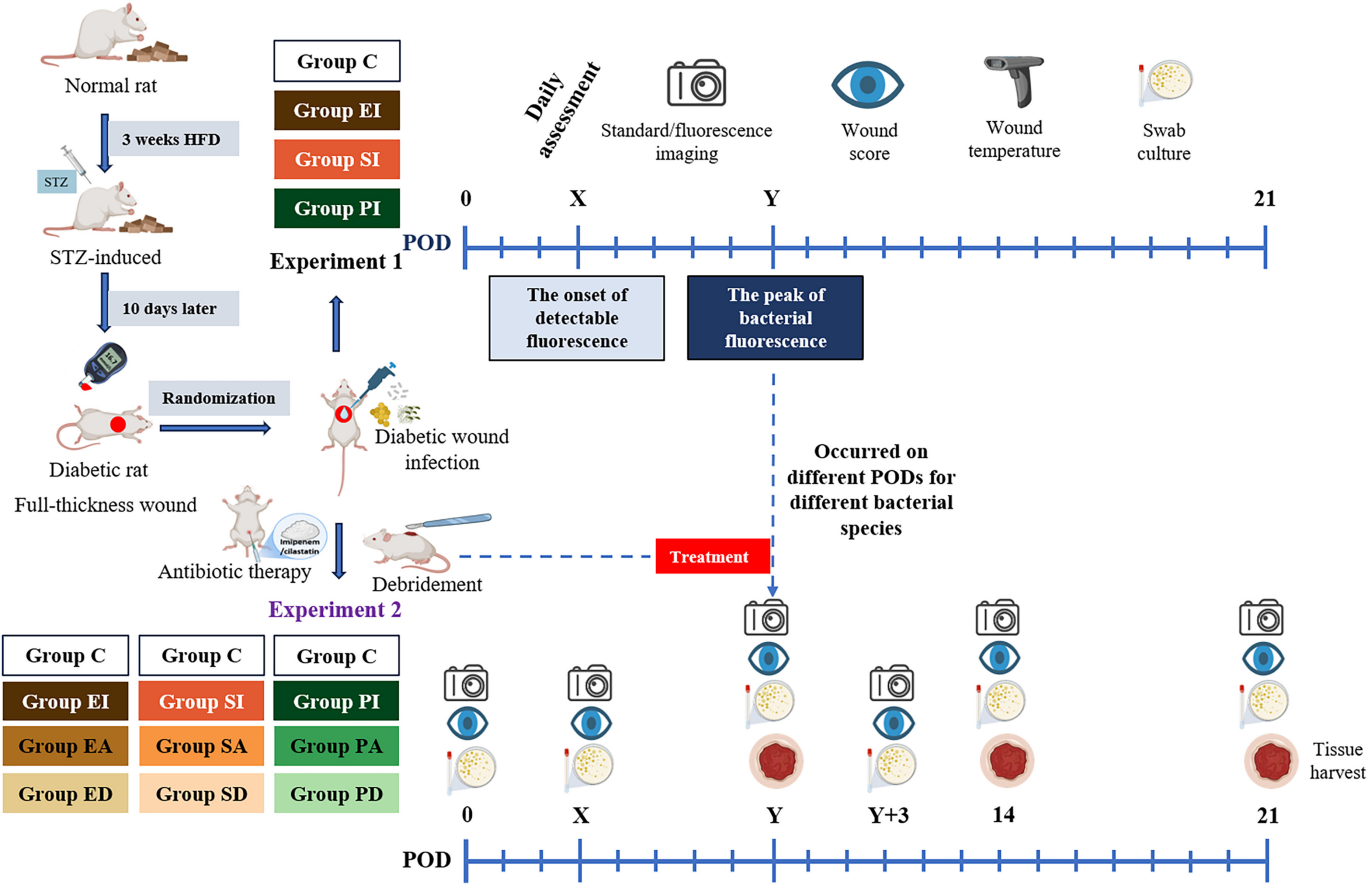
Schematic diagram of model establishment, experimental grouping, and time schedule for outcome measurements. POD X, the day when bacterial fluorescence signal was first observed; POD Y, the day corresponding to the peak fluorescence intensity, which marked the initiation of antimicrobial interventions. EI, *E. coli* infection; EA, *E. coli* infection + antibiotic; ED, *E. coli* infection + debridement; SI, *S. aureus* infection; SA, *S. aureus* infection + antibiotic; SD, *S. aureus* infection + debridement; PI, *P. aeruginosa* infection; PA, *P. aeruginosa* infection + antibiotic; PD, *P. aeruginosa* infection + debridement; HFD, high-fat diet; STZ, streptozotocin; POD, postoperative days.

To characterize bacterial fluorescence profiles in infected diabetic wounds and evaluate the impact of therapeutic interventions via fluorescence-based assessments, this study designed two experiments (Figure 1).

#### 2.8.1 Experiment 1: bacterial fluorescence dynamics in infected diabetic wounds

Diabetic rats were randomly assigned to four groups (*n* = 3): negative control (C), *E. coli* infection (EI), *S. aureus* infection (SI), and *P. aeruginosa* infection (PI) groups. Wound progression was monitored daily for up to 21 postoperative days (POD). Comprehensive wound assessments were conducted daily, including acquisition of standard and fluorescence images, wound scoring, wound temperature, and determination of bacterial burden using the swab sampling method.

#### 2.8.2 Experiment 2: effects of antimicrobial interventions on infected diabetic wounds

Based on the bacterial species and antimicrobial intervention strategy, rats were randomly assigned to: Control (*n* = 15), EI (*E. coli* infection only, *n* = 9), EA (*E. coli* infection + antibiotic, *n* = 9), ED (*E. coli* infection + debridement, *n* = 9), SI (*S. aureus* infection only, *n* = 9), SA (*S. aureus* infection + antibiotic, *n* = 9), SD (*S. aureus* infection + debridement, *n* = 9), PI (*P. aeruginosa* infection only, *n* = 9), PA (*P. aeruginosa* infection + antibiotic, *n* = 9), and PD (*P. aeruginosa* infection + debridement, *n* = 9) group. Imipenem/cilastatin (Merck Sharp & Dohme, New Jersey, United States) was administered intraperitoneally at a dose of 30mg/kg every 12 h for two consecutive days in the antibiotic groups. This administration route was chosen to avoid direct disruption of the wound site. In debridement groups, infected tissue was excised under fluorescence guidance after povidone–iodine disinfection. After debridement, fluorescence imaging was repeated to verify the complete removal of bacterial fluorescence. Over the following 2 days, fluorescence imaging was performed every 12 h; if bacterial fluorescence persisted, additional debridement was conducted until the fluorescence signals were no longer detec.

Key time points were defined based on Experiment 1: POD X, the day when bacterial fluorescence signal was first observed; POD Y, the day corresponding to the peak fluorescence intensity. In Experiment 2, antimicrobial interventions were initiated at POD Y. Wounds were systematically monitored and imaged on POD 0, X, Y, Y+3, 14, and 21. Tissue samples were collected on POD Y, 14, and 21 for bacteriological and histological analyses. Three rats per infected group were euthanized at each collection time point (*n* = 9 per group). Because POD Y differed among bacterial species, time-matched tissue sampling was performed in the group at the three pathogen-specific POD Y time points, as well as on POD 14 and 21. Consequently, the group C comprised 15 rats, with three rats allocated to each sampling time point.

### 2.9 Wound assessment

#### 2.9.1 Wound infection assessment

Wound severity was assessed using a clinically relevant scoring system based on previously published wound assessment methods and informed by key infection-related features included in the Infection component of the PEDIS classification system proposed by the International Working Group on the Diabetic Foot (IWGDF) (Gupta and Tyagi, 2021; Schaper, 2004). The scoring system encompassed eschar formation, edema/swelling, exudate, and the presence of pus or slough (Supplementary Table 1). Wound temperature was measured using a handheld infrared thermometer (Yuyue YT-3, Shanghai, China).

#### 2.9.2 Wound imaging and image analysis

Standard and fluorescence images were processed and quantified using ImageJ software (NIH, Bethesda, MD, United States). Wound area was quantified from standardized images and expressed as a percentage of the initial wound size using the following formula:


$$\mathrm{Wound}\mathrm{area}\left(\%\right)=A_{n}/A_{0}\times 100\%$$

where An represents the wound area on POD n and A0 the area on POD 0.

Fluorescence images were processed following established protocols with slight modification (Warncke et al., 2020; Wu et al., 2014). Briefly, fluorescence images were split into red-green-blue (RGB) channels. Red-channel signals were used for *S. aureus* and *E. coli*, and green-channel signals for *P. aeruginosa*. The bacterial colonies or wound beds were selected as the regions of interest (ROIs) (Supplementary Figure 3). The distribution (in pixels) and mean gray value of fluorescence within ROIs were quantified to reflect the fluorescence area and its intensity.

### 2.10 Wound tissue collection and processing

At each predetermined sampling time point (POD Y, 14, and 21), three rats per group were euthanized by gradual exposure to increasing concentrations of CO_2._ Following euthanasia, the entire wound together with the surrounding skin was harvested using a sterile 1-cm biopsy punch centered on the wound. Each wound specimen was bisected through the wound center. One half was fixed in 4% paraformaldehyde for histological analysis, whereas the other half was aseptically collected and processed for quantitative bacterial culture.

### 2.11 Bacterial burden

Bacterial burdens in wound exudate and tissue were assessed using selective media: MSA for *S. aureus*, EMB agar for *E. coli*, and *Pseudomonas* CFC agar for *P. aeruginosa*. Sterile swabs were rotated over the wound surface for 30 s and eluted in 1 mL PBS by vortexing. The resulting suspension was serially diluted in PBS to obtain final dilutions of 10^–3^, 10^–4^, and 10^–5^. From each dilution, 100μL was plated in triplicate onto selective media. For tissue-derived bacterial burden, the excised tissue was weighed and homogenized in 1mL of sterile PBS using a TissueMaster™ High-Throughput Tissue Homogenizer (Beyotime Biotechnology, Shanghai, China). The homogenate was then serially diluted to 10^–2^, 10^–3^, and 10^–4^ in PBS. Aliquots of 100μL from each dilution were plated in triplicate on selective agar plates. Plates were incubated aerobically at 37°C for 24 h, and colony counts were performed using the dilution plate that yielded 30–300 CFUs. Final bacterial burdens were expressed as log_10_ CFU/mL or log_10_ CFU/g.

### 2.12 Histopathology

Tissue specimens fixed in 4% paraformaldehyde were routinely processed, embedded in paraffin, and sectioned at 5 μm thickness. Sections were stained with H&E for histopathological evaluation. Briefly, H&E-stained sections were first subjected to quantitative histomorphometric analysis at 20 × magnification. Three representative sections per group (one section from each rat corresponding to the largest wound cross-sectional area) were analyzed for scar width, granulation tissue thickness, and epidermal thickness. Subsequently, these quantitative measurements and histopathological features were converted into histological scores according to the predefined scoring criteria described in Supplementary Table 2 (Dogan et al., 2009). Masson’s trichrome staining was performed to assess collagen deposition. Immunohistochemical staining for tumor necrosis factor-α (TNF-α, Abcam, Cambridge, United Kingdom) and transforming growth factor-β1 (TGF-β1, Abcam, Cambridge, United Kingdom) was performed using standard protocols. For the assessment of collagen deposition, inflammatory infiltration, and cytokine-positive cells, three non-overlapping high-power fields (HPFs) at 400 × magnification were randomly selected from each of three sections per animal, yielding a total of nine HPFs per animal for quantitative analysis. All evaluations were independently performed by two blinded pathologists.

### 2.13 Randomization and blinding

Diabetic rats were randomly assigned to experimental groups using a computer-generated randomization sequence. In Experiment 2, animals were randomly assigned to predefined postoperative sacrifice time points prior to longitudinal follow-up, and all animals were euthanized for tissue collection at their designated time points. All outcome assessments, including wound evaluation, image acquisition, bacterial burden quantification, and histopathological analysis, were performed by independent investigators blinded to group allocation.

### 2.14 Statistical analysis

The primary objective of Experiment 1 was to characterize the temporal dynamics of bacterial fluorescence, particularly the timing of peak fluorescence intensity. Accordingly, sample size estimation for this endpoint was performed using a precision-analysis approach, with a predefined estimation precision of within 1 day. A pilot study was conducted using three rats per group, and fluorescence imaging was performed at 12-h intervals (0.5 day). The minimum required sample size per group was subsequently calculated using the following formula:


$$n={\left(\frac{Z_{\alpha /2}\times \mathrm{SD}}{d}\right)}^{2}$$

Based on the results of the pilot study shown in Supplementary Table 3, Z_α/2_ = 1.96 for a 95% confidence level, the largest observed standard deviation was SD = 0.58, and the desired estimation precision was set at *d* = 1. Substituting these values into the formula yielded:


$$n={\left(\frac{1.96\times 0.58}{1}\right)}^{2}=1.29$$

Thus, the calculated minimum sample size was approximately two animals per group. Considering biological variability, potential longitudinal data loss, repeated-measures follow-up requirements, and animal welfare principles, three animals per group were ultimately included in the study.

All data were presented as mean ± standard deviation (SD). Data normality was assessed using Shapiro-Wilk test, and homogeneity of variances using Levene’s test. Continuous variables (e.g., wound area, glucose, cytokine levels) that met parametric assumptions were analyzed using Student’s *t*-test for two groups comparisons or two-way analysis of variance (ANOVA) followed by Tukey’s honestly significant difference (HSD) post hoc test for multiple comparisons. For data that did not meet normality criteria, the Kruskal–Wallis test was applied, followed by Dunn’s *post-hoc* test. Linear regression analysis was performed to evaluate the relationships among bacterial burden, fluorescence intensity, and fluorescence area. Statistical analyses were performed with SPSS version 22.0 (IBM, Armonk, NY, United States). A two-tailed *P* < 0.05 was considered statistically significant.

## 3 Results

### 3.1 Susceptibility to imipenem

Antimicrobial susceptibility testing demonstrated that all bacterial strains used in this study were uniformly susceptible to imipenem (Supplementary Figure 4).

### 3.2 *In vitro* visualization of bacterial fluorescence

We first characterized the fluorescence signatures of bacterial suspensions and colonies (Supplementary Figure 5). Under white light, all three bacterial suspensions appeared slightly turbid relative to LB broth, species-specific differences were not discernible. Upon excitation at 405 nm, both liquid and solid LB media exhibited faint green fluorescence. In liquid cultures, *E. coli* showed strong red fluorescence, whereas *S. aureus* showed comparatively weaker orange-red fluorescence at 24 h. By 48 h, however, the red fluorescence of *S. aureus* had markedly increased, surpassing that of *E. coli*. *P. aeruginosa* emitted bright green fluorescence at 24 h, which further intensified by 48 h. In solid cultures, colonies of *E. coli* and *S. aureus* became visible, but no red fluorescence was present at 12 h. By 24 h, both species exhibited strong red fluorescence, which subsequently declined over time. By contrast, *P. aeruginosa* colonies displayed detectable cyan fluorescence as early as 12 h, followed by a continuous increase in fluorescence intensity and lateral spread into the surrounding agar. The above results demonstrate that our constructed bacterial fluorescence imaging system can stably and effectively detect the characteristic fluorescence signals of the studied bacteria across different culture formats.

### 3.3 Successful induction of diabetes mellitus

Rats with fasting blood glucose levels exceeding 16.7 mmol/L were classified as having developed T2DM. At baseline, no statistically significant differences in blood glucose levels or body weights were observed among the experimental groups (*P* > 0.05) (Supplementary Table 4).

### 3.4 Longitudinal evaluation of wound infection in Experiment 1

#### 3.4.1 Temporal progression of wound infection and bacterial fluorescence

Representative standard and fluorescence images of diabetic wounds monitored over a 21-day period are shown in Supplementary Figure 6. In the control group, no signs of infection or bacterial fluorescence were observed. Skin tissue exhibited weak green fluorescence, and hair displayed bright green fluorescence, whereas the wound bed appeared dark and non-fluorescent. Bacterial fluorescence in the EI and SI groups manifested as red signals, whereas cyan fluorescence was observed in the PI group. In the EI, SI, and PI groups, bacterial fluorescence was first detected on POD 3, 2, and 1, respectively, when no clinical signs of infection were yet apparent. As time progressed, clinical signs of infection and bacterial fluorescence gradually intensified in each infected group. Wounds infected with *E. coli* showed marked serous exudation, *S. aureus* infections produced abundant purulent discharge with thick eschar formation, and *P. aeruginosa* infections were distinguished by dense, biofilm-like slough with extensive surface deposition.

**FIGURE 2:**
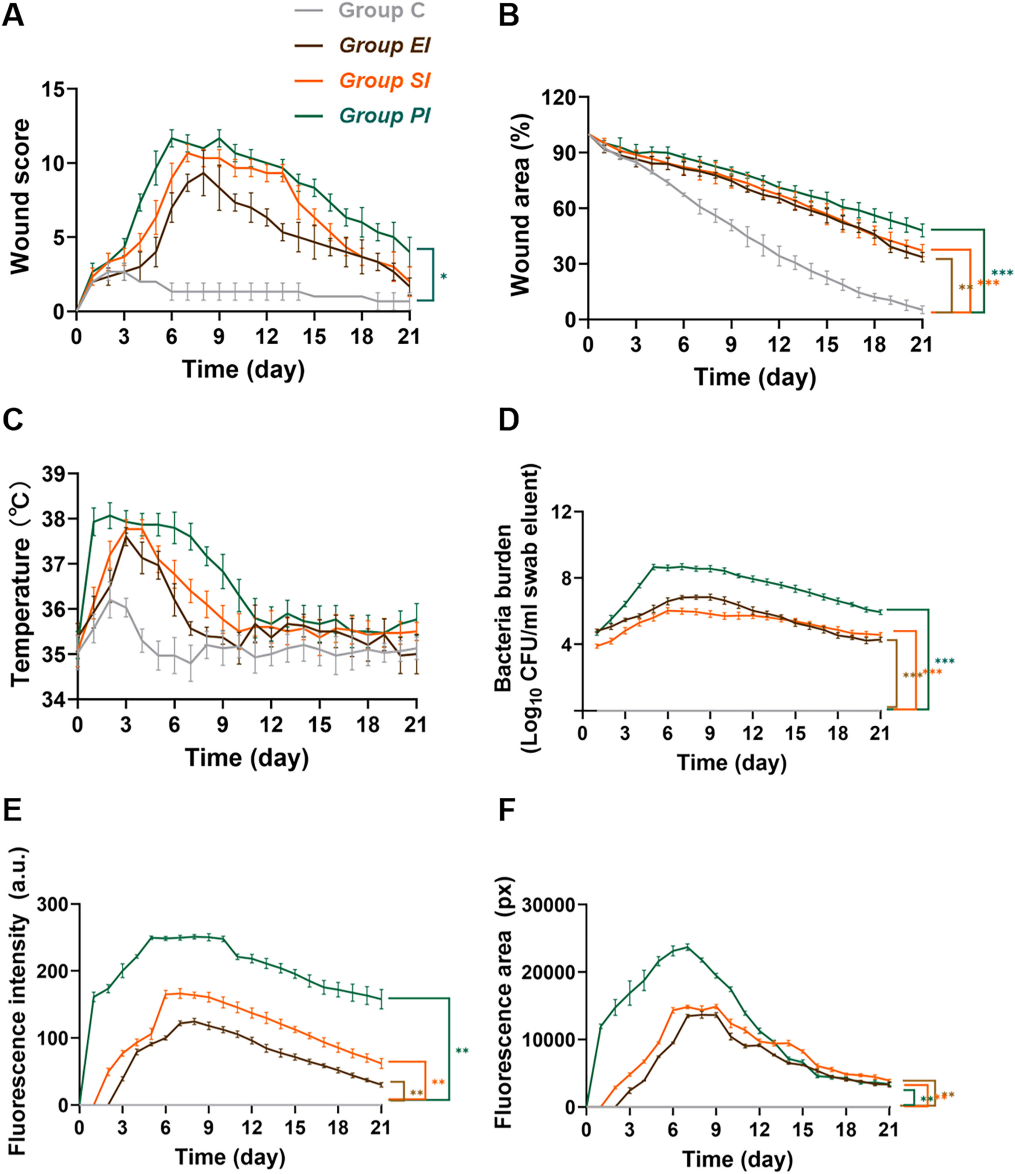
Longitudinal assessment of wound characteristics. **(A)** Time-course measurements of wound score, **(B)** wound area, **(C)** wound temperature, **(D)** bacterial burden, **(E)** fluorescence intensity, and **(F)** fluorescence area in infected and non-infected wounds over a 21-day period. Data are presented as mean ± SD (*n* = 3). Comparisons between each infected group and the control group were performed using Student’s *t*-test with Benjamini–Hochberg false discovery rate (FDR) correction. *P < 0.05, **P < 0.01, ***P < 0.001. For clarity, statistical significance is shown only for POD 21, results for all time points are presented in Supplementary Figure 7.

The characteristics of wounds were quantitatively assessed and compared (Figure 2 and Supplementary Figure 7). From POD 1 onward, the bacterial burden in all infected groups remained significantly higher than that of the control group. Wound temperature increased only during the acute phase of infection. Therefore, temperature was not measured in Experiment 2. Bacterial fluorescence became detectable prior to clinical signs, appearing on POD 3 (EI group), POD 2 (SI group), and POD 1 (PI group). Signals peaked on POD 7 (EI group), POD 6 (SI group), and POD 5 (PI group), respectively. The EI, SI, and PI groups exhibited higher wound scores than the control group from POD 6 to 17, from POD 4 to 20, and from POD 3 to 21, respectively. Infection also delayed wound closure, with all infected wounds exhibiting larger wound areas than the control group from POD 4 or 5 onward.

#### 3.4.2 Correlation of bacterial fluorescence with swab-derived bacterial burden

Correlation analysis revealed that fluorescence intensity showed strong correlations with swab-derived bacterial burden across all pathogens. Fluorescence area correlated strongly in *E. coli* and *S. aureus*, but only moderately in *P. aeruginosa* (Table 1). Therefore, fluorescence intensity was selected for quantifying bacterial fluorescence in experiment 2.

**TABLE 1:** Pearson’s correlation matrix between bacterial fluorescence parameters and swab-derived bacterial burden.

Parameter	Pearson correlation r	*P*-value
E. coli		
Fluorescence intensity	0.9505	< 0.0001
Fluorescence area	0.8847	< 0.0001
S. aureus		
Fluorescence intensity	0.9634	< 0.0001
Fluorescence area	0.9426	< 0.0001
P. aeruginosa		
Fluorescence intensity	0.9226	< 0.0001
Fluorescence area	0.5981	0.0042

### 3.5 Effects of antimicrobial interventions

#### 3.5.1 Treatment effects on fluorescence, bacterial burden, and wound healing

**FIGURE 3:**
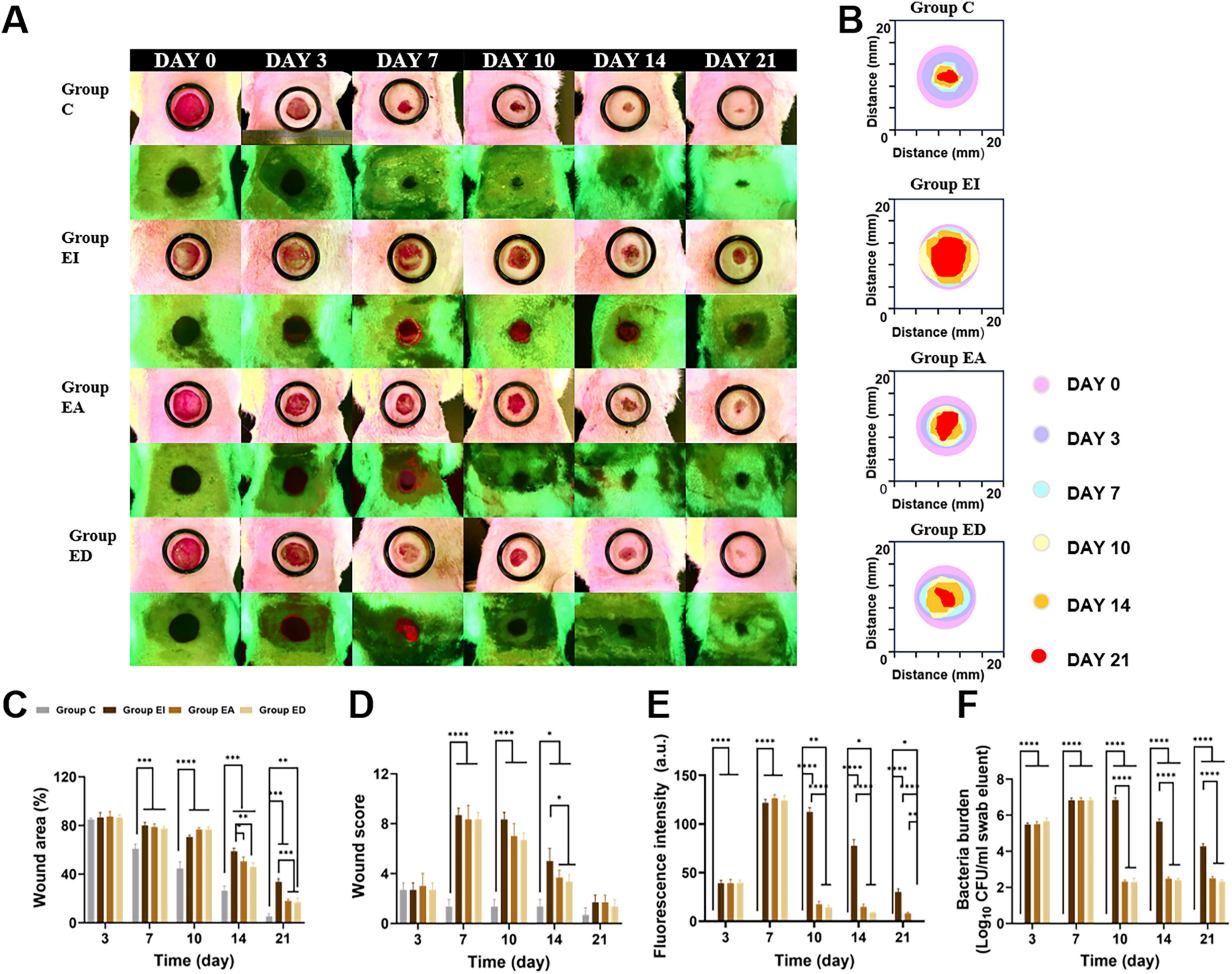
Therapeutic efficacy in *Escherichia coli*–infected diabetic wounds. **(A)** Representative standard and fluorescence images. **(B)** Wound tracings with different treatments. Quantitative comparison of wound area **(C)**, wound score **(D)**, bacterial fluorescence intensity **(E)**, and swab-derived bacterial burden **(F)** among the experimental groups. Data are presented as mean ± SD (*n* = 3). Statistical analyses were performed using two-way ANOVA followed by Tukey’s multiple-comparisons test.*P < 0.05, **P < 0.01, ***P < 0.001,*⁣*⁣*⁣*P < 0.0001.

**FIGURE 4:**
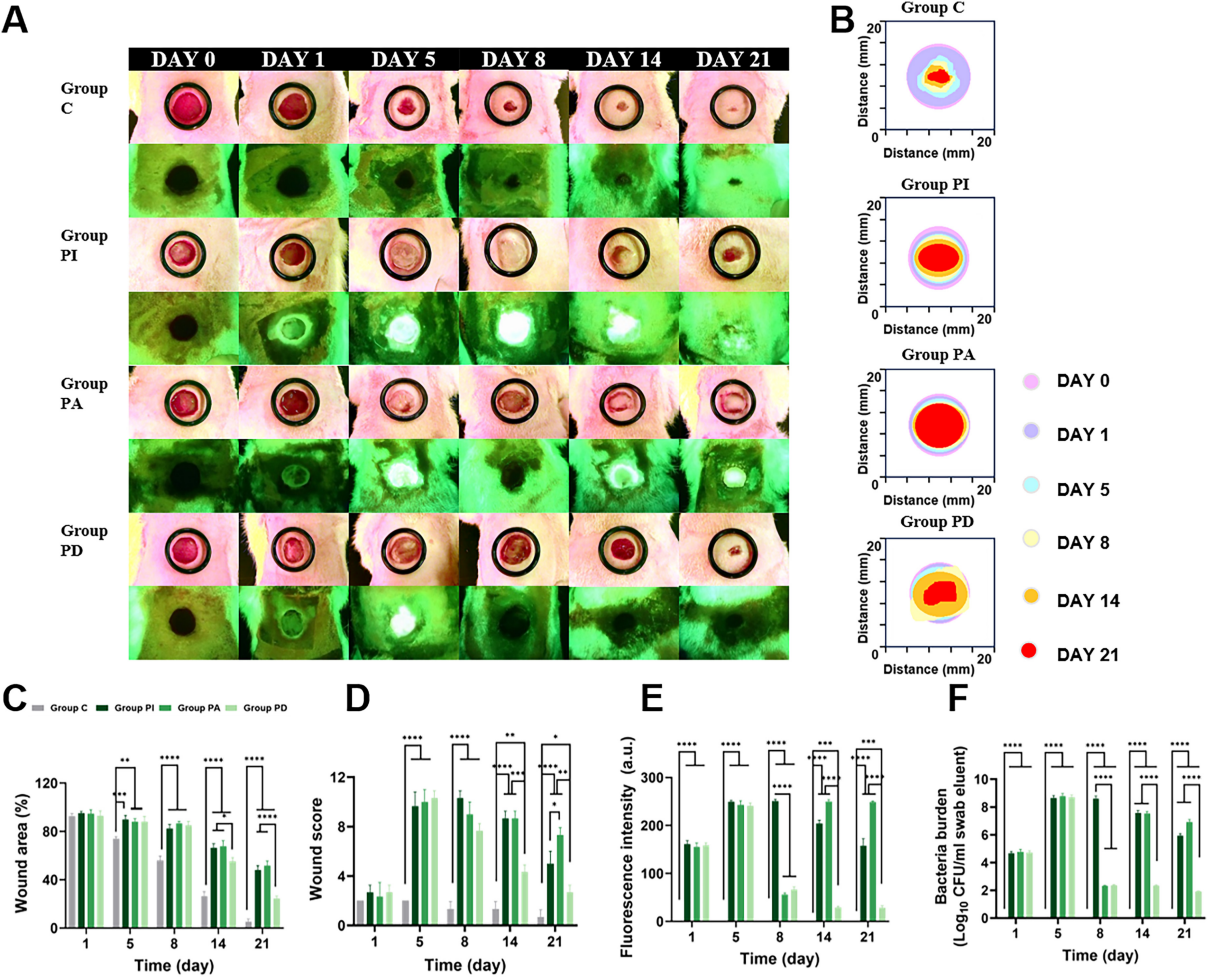
Therapeutic efficacy in *Pseudomonas aeruginosa*–infected diabetic wounds. **(A)** Representative standard and fluorescence images. **(B)** Wound tracings with different treatments. Quantitative comparison of wound area **(C)**, wound score **(D)**, bacterial fluorescence intensity **(E)**, and swab-derived bacterial burden **(F)** among the experimental groups. Data are presented as mean ± SD (*n* = 3). Statistical analyses were performed using two-way ANOVA followed by Tukey’s multiple-comparisons test.*P < 0.05, **P < 0.01, ***P < 0.001,*⁣*⁣*⁣*P < 0.0001.

Antimicrobial interventions were initiated at the peak fluorescence day to systematically evaluate their effects on fluorescence intensity, bacterial burden, and wound status (Figures 3, 4 and Supplementary Figure 8). In wounds infected with *E. coli* or *S. aureus*, both antibiotic and debridement markedly reduced bacterial fluorescence and bacterial burden, and decreased wound scores and wound areas, with comparable outcomes on POD 14 and POD 21. In contrast, in *P. aeruginosa* infection, antibiotic treatment produced only transient reductions in fluorescence and bacterial burden, followed by rapid recurrence. By POD 14 and POD 21, fluorescence intensity, bacterial burden, wound score, and residual wound area in the PA group were significantly higher than those in the PD group. Debridement achieved sustained control and significantly improved wound healing outcomes.

#### 3.5.2 Histological responses to antimicrobial interventions

**FIGURE 5:**
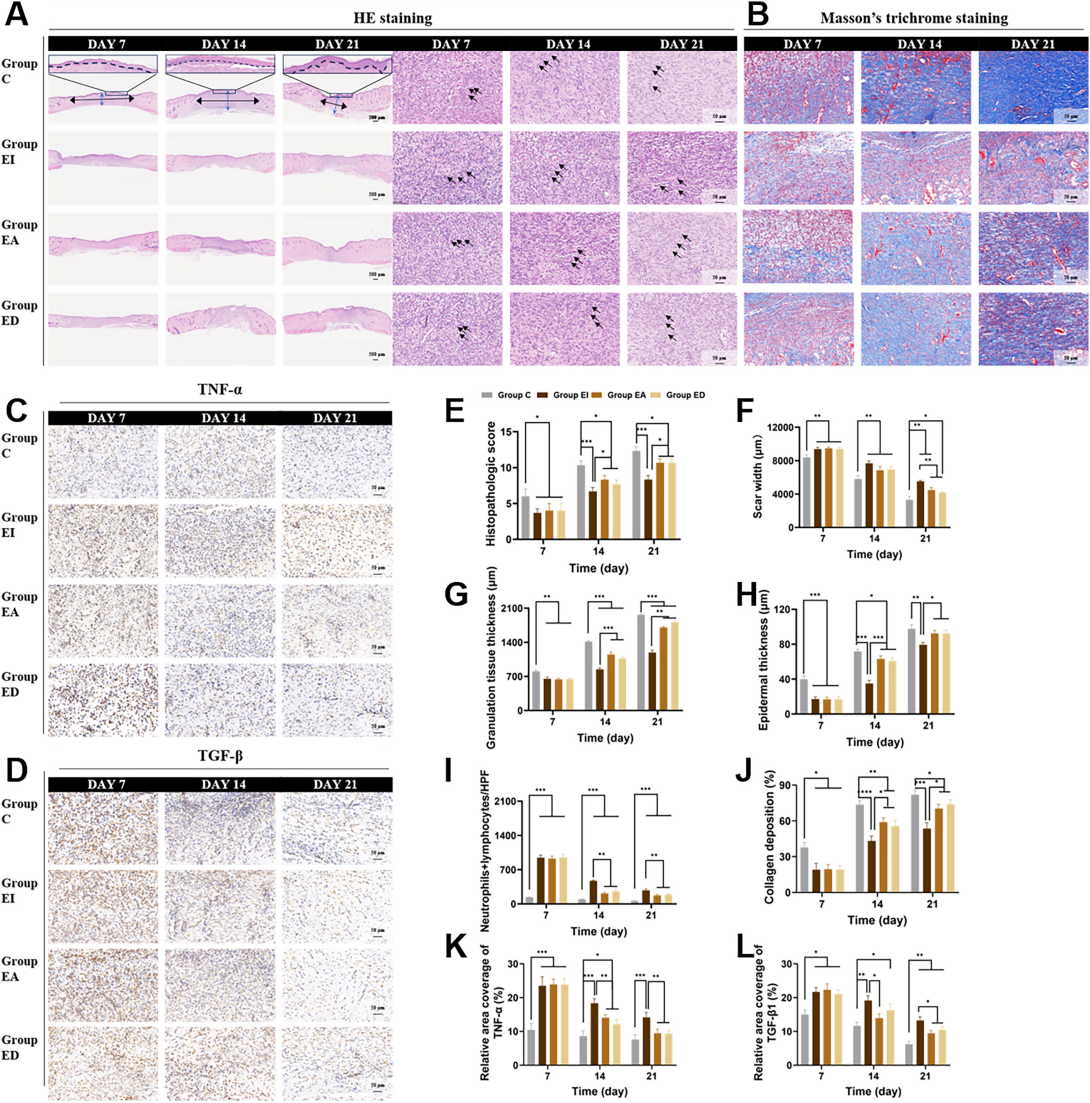
Histological evaluation in *Escherichia coli*–infected diabetic wounds with different treatments. **(A)** H&E staining, **(B)** Masson’s trichrome staining, and immunohistochemical staining for **(C)** TNF-α and **(D)** TGF-β1 in wound tissue sections. Quantitative comparison of histopathological scores **(E)**, scar width **(F)**, granulation tissue thickness **(G)**, epithelial thickness **(H)**, inflammatory cell infiltration **(I)**, collagen deposition **(J)** TNF-α **(K)** and TGF-β1 **(L)** across different treatment groups. Black double-headed arrows indicate the scars. Blue double-headed arrows indicate the granulation tissues. Black dashed lines delineate the regenerated epithelium. Black arrows indicate inflammatory cells. Data are presented as mean ± SD (*n* = 3). Statistical analyses were performed using two-way ANOVA followed by Tukey’s multiple-comparisons test.*P < 0.05, **P < 0.01, ***P < 0.001,*⁣*⁣*⁣*P < 0.0001.

**FIGURE 6:**
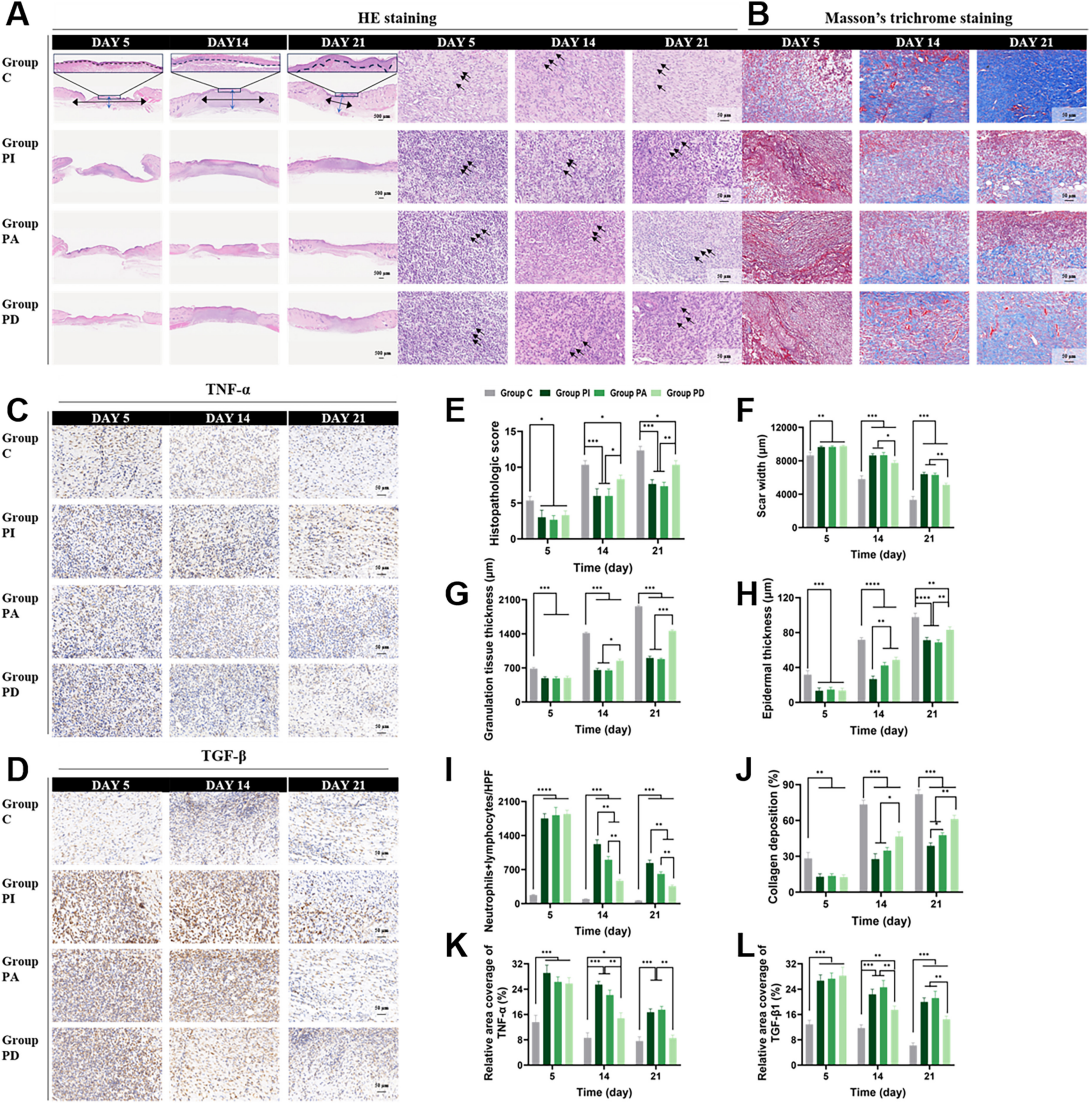
Histological evaluation in *Pseudomonas aeruginosa*–infected diabetic wounds with different treatments. **(A)** H&E staining, **(B)** Masson’s trichrome staining, and immunohistochemical staining for **(C)** TNF-α and **(D)** TGF-β1 in wound tissue sections. Quantitative comparison of histopathological scores **(E)**, scar width **(F)**, granulation tissue thickness **(G)**, epithelial thickness **(H)**, inflammatory cell infiltration **(I)**, collagen deposition **(J)** TNF-α **(K)** and TGF-β1 **(L)** across different treatment groups. Black double-headed arrows indicate the scars. Blue double-headed arrows indicate the granulation tissues. Black dashed lines delineate the regenerated epithelium. Black arrows indicate inflammatory cells. Data are presented as mean ± SD (*n* = 3). Statistical analyses were performed using two-way ANOVA followed by Tukey’s multiple-comparisons test.*P < 0.05, **P < 0.01, ***P < 0.001,*⁣*⁣*⁣*P < 0.0001.

Infected wounds exhibited impaired healing characterized by wider scar width, thinner regenerating epithelium, reduced granulation tissue thickness, increased inflammation, decreased collagen deposition, and significantly lower histopathological scores compared with the control group (Figures 5, 6 and Supplementary Figure 9). Both treatments improved outcomes in *E. coli* and *S. aureus*. However, in wounds infected with *P. aeruginosa*, were observed among exhibited narrower scar widths, thicker regenerating epithelium, greater granulation tissue thickness, reduced inflammation, and higher histopathological scores, demonstrating superior efficacy in promoting wound healing compared with antibiotic therapy.

In the control group, the expressions of TNF-α and TGF-β1 remained at consistently low levels throughout the observation period. Infection induced marked increases in both cytokines. In wounds infected with *E. coli* and *S. aureus*, either antibiotic therapy or debridement effectively reduced TNF-α and TGF-β1, indicating attenuation of local inflammation and promotion of tissue repair. In contrast, in wounds infected with *P. aeruginosa*, persistently elevated TNF-α and TGF-β1 levels were still observed on POD 14 and 21 in the PA group, suggesting that antibiotic therapy was insufficient to fully control inflammation or restore normal wound healing dynamics.

### 3.6 Correlation between fluorescence intensity and bacterial burden

Correlation analyses were performed to elucidate the relationships among swab-derived bacterial burden, tissue-derived bacterial burden, and bacterial fluorescence (Supplementary Figure 10). A strong positive correlation was observed between swab- and tissue-derived bacterial burden across all species. Furthermore, linear regression analysis showed that fluorescence intensity was more strongly correlated with swab-derived than with tissue-derived bacterial burden.

## 4 Discussion

Timely detection and continuous monitoring of DFIs are crucial to prevent disease progression, optimize therapeutic strategies, and improve prognosis (Lipsky, 2016; Saltoglu et al., 2023). Using a rat model of diabetic wound infection, our findings suggest that bacterial fluorescence precedes overt CSS, with fluorescence intensity showing a strong positive correlation with bacterial burden and high sensitivity to ongoing infection and delayed wound healing. Collectively, these results suggest that bacterial fluorescence may serve as an early, non-invasive biomarker for detecting infection, tracking disease progression, and predicting wound healing.

T2DM accounts for approximately 90% of all diabetes cases (Ahmad et al., 2022). The T2DM experimental model used in this study has been widely applied and is characterized by hyperglycemia, obesity-associated metabolic dysfunction, and insulin resistance (Furman, 2015; Reed et al., 2000). The resulting wound microenvironment is characterized by prolonged hyperglycemia, severe hypoxia, elevated reactive oxygen species (ROS), chronic inflammation, and an abnormally alkaline pH, all of which contribute to impaired wound healing and increased susceptibility to infection (Zhao et al., 2016; Zhou et al., 2026). Accordingly, this model is widely used in studies of diabetic wound infection and the evaluation of antimicrobial therapies due to its considerable clinical relevance and translational value. *S. aureus*, *E. coli* and *P. aeruginosa* are among the most common pathogens implicated in DFIs (Citron et al., 2007; Macdonald et al., 2021). In previous studies, inoculation doses ranging from 10^3^ to 10^8^ CFU of *E. coli, S. aureus*, and *P. aeruginosa* have commonly been used, varying according to bacterial species, wound type, host immune status, and inoculation strategy (Brandenburg et al., 2019; Moslemi et al., 2012; Ramírez-Tapia et al., 2025). Previous fluorescence imaging studies have predominantly used nude mouse models with relatively high bacterial inoculation doses (10^7^–10^9^ CFU), primarily to ensure rapid infection establishment (Lopez et al., 2021; Warncke et al., 2020; Wu et al., 2014). In contrast, the present study employed a substantially low inoculum (10^3^ CFU), a dose commonly used in infection-prone wound models, including burn and diabetic wounds. Importantly, despite the low inoculum, the model consistently developed stable and reproducible wound infections, as evidenced by sustained bacterial burdens exceeding the infection-associated threshold (10^5^ CFU/g tissue), pronounced signs of infection, and significantly delayed wound healing. Compared with high-inoculum infection models established in nude mice, the present diabetic rat model more closely recapitulates the pathophysiological processes underlying clinical wound infections. Rather than relying on an overwhelming bacterial challenge, infection was established through biologically relevant host–pathogen interactions involving the interplay between bacterial virulence and host immune defenses. The bacterial fluorescence peak (POD 5–7), POD 14, and POD 21 were selected as key observation time points based on temporal infection dynamics reported in relevant experimental wound infection models, together with established principles of wound healing progression (Rahim et al., 2017; Zhang et al., 2025). These time points corresponded to the acute infection stage, persistent infection stage, and late-stage healing phase, respectively. Longitudinal assessment across these representative stages enabled characterization of the dynamic continuum between active infection and tissue repair, thereby facilitating integrated evaluation of bacterial burden, wound phenotype evolution, and healing progression in diabetic infected wounds. Consequently, this non-lethal and physiologically relevant model may provide a more robust and clinically relevant platform for the longitudinal monitoring of diabetic wound infections.

Fluorescence intensity and fluorescence area were both strongly correlated with bacterial burden in *E. coli* and *S. aureus* infections. In contrast, in *P. aeruginosa* infection, fluorescence intensity remained strongly correlated with bacterial burden, whereas fluorescence area showed only a moderate correlation. This discrepancy may be attributed to the biological properties of pyoverdine, which, although synthesized intracellularly, is rapidly secreted into the extracellular environment for iron acquisition (Imperi et al., 2009). In addition, pyoverdine production is influenced by the wound microenvironment. Iron limitation, alkaline pH, and biofilm-associated bacterial activity may further enhance pyoverdine secretion (Butaitė et al., 2018; Capatina et al., 2022; Kang et al., 2017). Consequently, extracellular accumulation of pyoverdine may lead to an overestimation of bacteria when quantified by fluorescence area. Therefore, these findings suggest that, in clinical *P. aeruginosa*-infected wounds, fluorescence area should not be used in isolation to determine infection extent, assess treatment response, or guide debridement margins, but rather interpreted in conjunction with fluorescence intensity, CSS, and other microbiological assessments. Meanwhile, pyoverdine-associated fluorescence may reflect metabolically active and virulence-associated bacterial states, thereby providing complementary information regarding infection activity. Further studies are warranted to clarify the clinical significance of fluorescence area in monitoring *P. aeruginosa* infections. Importantly, bacterial fluorescence was detectable prior to the onset of CSS, with a detection threshold of approximately 10^4^ CFU/g, consistent with previous clinical observations (Rennie et al., 2017). Given the well-established association between bacterial burdens ≥ 10^5^ CFU/g and clinical infection, fluorescence imaging may offer a valuable tool for the detection of subclinical infections (Bowler et al., 2001; Farhan and Jeffery, 2021).

Antibiotic therapy and debridement constitute the two fundamental pillars in the management of DFIs, both effectively reducing bacterial burden and attenuating inflammatory cascades (Dayya et al., 2022; Nikoloudi et al., 2018). In the present study, imipenem/cilastatin was selected as a clinically relevant systemic antibiotic because of its broad-spectrum activity against the pathogens investigated (*E. coli*, *S. aureus*, and *P. aeruginosa*) and its established role in the treatment of moderate-to-severe DFIs (Selva Olid et al., 2015). Current IWGDF/IDSA guidelines recognize carbapenems as important therapeutic options, particularly when *P. aeruginosa* or ESBL-producing organisms are suspected (Senneville et al., 2024). Furthermore, all bacterial strains used in this study were confirmed to be susceptible to imipenem/cilastatin *in vitro*. In addition, debridement mechanically removes necrotic tissue and biofilms, thereby improving tissue perfusion and facilitating biofilm-associated infection control. A prospective clinical study showed that a single debridement can lower wound bacterial burdens from 10^6^ to 10^8^ CFU/g to below 10^4^ CFU/g, while repeated debridement can further decrease bacterial burdens to less than 10^2^ CFU/g (Yarets, 2017). Our study demonstrated that fluorescence-guided debridement significantly reduced bacterial burden and inflammatory responses across all infection models, ultimately accelerating wound healing. These results, together with previous reports, suggest fluorescence-guided debridement as a promising strategy for infection control. In contrast, antibiotic therapy showed pathogen-dependent efficacy. All tested bacterial strains were confirmed to be susceptible to the selected antibiotics *in vitro*. While antibiotic therapy and debridement achieved comparable outcomes in *E. coli* and *S. aureus* infections, antibiotic therapy was significantly less effective in *P. aeruginosa*. Both interventions effectively controlled infection at early stages. However, bacterial signal rapidly rebounded in the antibiotic-treated wounds, accompanied by delayed wound healing, whereas debridement achieved sustained bacterial suppression and improved tissue repair. This discrepancy is likely driven by the unique infectious milieu of *P. aeruginosa* and its interaction with imipenem. Virulence factor-mediated tissue necrosis, excessive exudation, and biofilm formation resulting from extracellular polymeric substance (EPS) production collectively create a physical and biochemical barrier that limits antibiotic penetration and reduces local drug exposure (Brandenburg et al., 2019; Hou et al., 2024). Notably, subinhibitory imipenem exposure may further induce adaptive biofilm responses, including enhanced AmpC β-lactamase production and increased alginate biosynthesis (Bagge et al., 2004). These adaptations can reinforce biofilm integrity, promote the persistence of biofilm-embedded bacteria, and increase antimicrobial tolerance (Hengzhuang et al., 2012). Consequently, residual bacteria may survive within this protected microenvironment, proliferate during the post-treatment period, and contribute to infection recurrence. By contrast, fluorescence imaging enables visualization of both planktonic bacteria and biofilm-associated structures (Lopez et al., 2021). Fluorescence-guided debridement allows more precise removal of bacterial aggregates and biofilm-associated tissues, thereby disrupting the microenvironment that sustains persistent infection. This mechanism may explain its superior efficacy in controlling *P. aeruginosa* infection in the present study. Collectively, our findings, together with previous evidence, underscore the importance of debridement in the management of chronic wounds with suspected *P. aeruginosa* infection and biofilm involvement (Malone and Swanson, 2017).

Current clinical practice relies primarily on tissue biopsy and swab culture for pathogen identification. While tissue biopsy remains the gold standard, its invasiveness limits repeated use. Swab culture provides a less invasive alternative but is influenced by sampling variability. Both culture-based approaches require prolonged incubation and are susceptible to contamination or culture failure. In line with previous studies, we observed a strong positive correlation between swab- and tissue-derived bacterial burdens (Haalboom et al., 2018). Furthermore, we found both approaches exhibited robust correlations with fluorescence intensity throughout the natural course of infection and following antimicrobial treatment. Fluorescence intensity correlated more strongly with swab-derived than with tissue-derived bacterial burden. This difference may be explained by the limited penetration of ultraviolet light into tissue, which attenuates fluorescence signals from deeper bacterial populations and weakens the observed association. Collectively, the strong and consistent relationships among fluorescence intensity, swab bacterial burden, and tissue bacterial burden support the potential use of fluorescence imaging as a non-invasive surrogate marker of wound bioburden. Unlike conventional microbiological culture, which typically requires several days for pathogen identification, fluorescence imaging may provide non-contact, rapid, real-time, point-of-care visualization of bacterial activity and spatial distribution. It may therefore help transform diabetic wound infection from a state that is “invisible, delayed, and imprecisely characterized” into one that is “visible, immediate, and more accurately assessed.” This may facilitate earlier clinical decision-making, optimized wound sampling, targeted debridement, and timely adjustment of antimicrobial therapy, while also offering valuable opportunities for patient education and improved engagement in wound management. For example, real-time visualization of bacterial burden and its response to treatment may help clinicians communicate infection status more intuitively to patients, improve adherence to treatment regimens, and support shared decision-making.

Several limitations should be acknowledged. First, fluorescence imaging may fail to detect non-fluorescent organisms, including *Enterococcus faecalis* and *Streptococcus agalactiae*, as well as certain fungi (Jones et al., 2020). Second, the relatively small sample size may limit the statistical robustness and generalizability of the findings. Therefore, larger-scale validation studies are warranted in future work. Third, species differentiation based solely on fluorescence remains limited. Chronic wounds are frequently polymicrobial, whereas current fluorescence imaging primarily distinguishes pyoverdine-associated fluorescence from porphyrin-associated fluorescence and cannot provide definitive species identification or antimicrobial resistance information. Thus, fluorescence imaging should currently be considered a complementary adjunct to conventional microbiological diagnostics rather than a replacement for culture-based assessment. Fourth, comprehensive metabolic characterization was not performed in the present study. This may introduce inter-individual variability in metabolic status and partially limit the robustness and translational relevance of the findings to human type 2 diabetic wounds. In addition, T1DM and T2DM differ in immune regulation, inflammatory responses, and wound microenvironmental characteristics, all of which may influence bacterial colonization and infection progression (Sun et al., 2025). Therefore, caution is warranted when extrapolating the present findings to other diabetic phenotypes. Finally, clinical implementation of fluorescence imaging may require dedicated imaging equipment and standardized operator training, which could increase initial costs and limit accessibility in primary healthcare settings. Future integration of spectral analysis, quantitative fluorescence characterization, and artificial intelligence-assisted image interpretation may further improve pathogen differentiation, antimicrobial resistance-related information acquisition, and interpretation accuracy, thereby expanding the clinical utility of this technology.

## 5 Conclusion

In summary, this preclinical study characterizes the fluorescence properties of three clinically relevant wound pathogens in a diabetic rat model. Our findings indicate that bacterial fluorescence correlates closely with bacterial burden and may provide a non-invasive, repeatable approach for early, real-time assessment of infection and treatment response. Further clinical validation through well-designed prospective studies is warranted to determine its potential applicability in the management of DFIs.

## Data Availability

The raw data supporting the conclusions of this article will be made available by the authors, without undue reservation.
