# Supplementary material for: Real-time detection and monitoring of bacteria in diabetic wounds using bacterial fluorescence
Source: Front Microbiol. 2026 Jul 3;17:1867420. doi: 10.3389/fmicb.2026.1867420 (PMC13375788; doi:10.3389/fmicb.2026.1867420)
Supplement: Supplementary file 1 [file Data_Sheet_1.docx]

Supplementary Material

**Supplementary Table 1.** Visual wound bed scoring system.

**Supplementary Table 2.** Histological scoring criteria for wound healing assessment.

**Supplementary Table 3.** Temporal characteristics of bacterial fluorescence in different groups

**Supplementary Table 4.** Baseline of blood glucose and body mass in the groups.

**Supplementary Figure 1.** Bacterial fluorescence imaging system diagram.

**Supplementary Figure 2.** Standardization curve for CFU/mL versus OD_600_.

**Supplementary Figure 3.** Preprocessing of fluorescence images.

**Supplementary Figure 4.** Antimicrobial susceptibility of the bacterial strains to imipenem

**Supplementary Figure 5.** In vitro characterization of bacterial fluorescence.

**Supplementary Figure 6.** Representative standard and fluorescence images of diabetic wound infection over a 21-day period.

**Supplementary Figure 7.** Comparison of wound characteristics between infected and non-infected wounds**.**

**Supplementary Figure 8.** Therapeutic efficacy in *Staphylococcus aureus*–infected diabetic wounds.

**Supplementary Figure 9.** Histological evaluation in *Staphylococcus aureus*–infected diabetic wounds with different treatments.

**Supplementary Figure 10.** Bacterial burdens quantified from wound swabs and tissues and their correlations with fluorescence intensity.^[[1]](#endnote-1)^

**Supplementary Table 1.** Visual wound bed scoring system

| **Feature of wound bed** | **Wound bed scores** | | | |
| --- | --- | --- | --- | --- |
|  | 0 | 1 | 2 | 3 |
| **Eschar formation** | No | Very thin eschar localized at the wound edge | Moderate eschar covering part of the wound bed | Thick, extensive eschar covering most or all of the wound bed |
| **Oedema/swelling** | No | Mild swelling localized to wound margins | Moderate swelling involving surrounding tissue | Severe, widespread swelling possibly with firmness or tenderness |
| **Fluid discharge** | No | Minimal clear or serous discharge | Moderate turbid or yellow discharge | Heavy, purulent or foul-smelling discharge |
| **Pus/slough** | No | Slight slough or pus limited to wound margins | Moderate presence on parts of the wound bed | Extensive thick slough or pus, possibly with abscess formation |

**Supplementary Table 2. Histological scoring criteria for wound healing assessment**

| **Parameter** | **Score Range** | **Scoring Criteria** |
| --- | --- | --- |
| **Re-epithelialization** | 0–4 | 0 = No closure 1 ≤ 30% coverage 2 = 31–60% coverage 3 = 61–99% coverage 4 = Complete re-epithelialization by keratinocytes |
| **Granulation Tissue Formation^*^** | 0–3 | 0 = Not observed 1 = Thin granulation layer 2 = Moderate granulation 3 = Thick granulation layer |
| **Cellular Infiltration** | 0–3 | 1 = Few cells 2 = Moderate number of cells 3 = Many cells (Only fibroblasts and macrophages were counted; polymorphonuclear cells and lymphocytes were excluded) |
| **Neovascularization** | 0–3 | 0 = 0–4 capillaries per wound section 1 = 5–14 capillaries 2 = 15–24 capillaries 3 ≥ 24 capillaries (Only mature capillaries containing erythrocytes were included) |

^*^For granulation tissue scoring, the granulation tissue thickness of the control group at POD 21 was used as the reference value. Thicknesses corresponding to 0–1/3, 1/3–2/3, and >2/3 of the reference value were assigned scores of 1 (thin granulation layer), 2 (moderate granulation), and 3 (thick granulation layer), respectively.

**Supplementary Table 3.** Temporal characteristics of bacterial fluorescence in different groups

| Group | Detectable fluorescence onset time (day)  （n = 3） | Peak fluorescence time (day)  （n = 3） |
| --- | --- | --- |
| **Negative control** | Not detected | Not detected |
| ***E. coli*** | 3.25 ± 0.29 | 7.50 ± 0.58 |
| ***S. aureus*** | 2.50 ± 0.58 | 6.00 ± 0.50 |
| *P. aeruginosa* | 1.00 ± 0.00 | 4.75 ± 0.29 |

Data are presented as mean ± SD.

**Supplementary Table 4.** Baseline of blood glucose and body mass in the groups

| **Experiment** | Group | N | Blood glucose (mmol/L) | Body weight (g) |
| --- | --- | --- | --- | --- |
| **Experiment 1** |  |  |  |  |
|  | Control | 3 | 23.11 ± 2.72 | 424.67 ± 21.78 |
|  | EI | 3 | 22.87 ± 1.64 | 422.33 ± 18.88 |
|  | SI | 3 | 24.29 ± 2.11 | 425.67 ± 19.55 |
|  | PI | 3 | 23.03 ± 1.62 | 423.33 ± 18.15 |
| **Experiment 2** |  |  |  |  |
| **Cluster 1**  **(*E. coli* series groups)** |  |  |  |  |
|  | Control | 9 | 24.49 ± 2.81 | 423.11 ± 18.92 |
|  | EI | 9 | 23.40 ± 2.19 | 430.11 ± 17.98 |
|  | EA | 9 | 22.95 ± 2.04 | 432.67 ± 18.84 |
|  | ED | 9 | 23.22 ± 2.09 | 428.67 ± 20.11 |
| **Cluster 2**  **(*S. aureus* series groups)** |  |  |  |  |
|  | Control | 9 | 25.34 ± 2.08 | 427.11 ± 19.34 |
|  | SI | 9 | 23.31 ± 1.99 | 425.33 ± 16.02 |
|  | SA | 9 | 23.36 ± 2.12 | 429.11± 17.14 |
|  | SD | 9 | 23.07 ± 2.04 | 427.11 ± 17.14 |
| **Cluster 3**  **(*P. aeruginosa***  **series groups)** |  |  |  |  |
|  | Control | 9 | 24.78 ± 2.30 | 424.22 ± 17.52 |
|  | PI | 9 | 22.83 ±2.01 | 428.89 ± 19.48 |
|  | PA | 9 | 22.58 ± 2.42 | 428.78 ± 20.64 |
|  | PD | 9 | 23.38 ± 2.02 | 426.00 ± 15.62 |

No statistically significant differences in blood glucose and body weight were observed among the four groups in Experiment 1 (*P* > 0.05). In Experiment 2, no statistically significant differences were observed among groups within each cluster (*P* > 0.05). Clusters represent series of interventions based on bacterial strain. EI, *E. coli* infection; EA, *E. coli* infection + antibiotic; ED, *E. coli* infection + debridement; SI, *S. aureus* infection; SA, *S. aureus* infection + antibiotic; SD, *S. aureus* infection + debridement; PI, *P. aeruginosa* infection; PA, *P. aeruginosa* infection + antibiotic; PD, *P. aeruginosa* infection + debridemen.

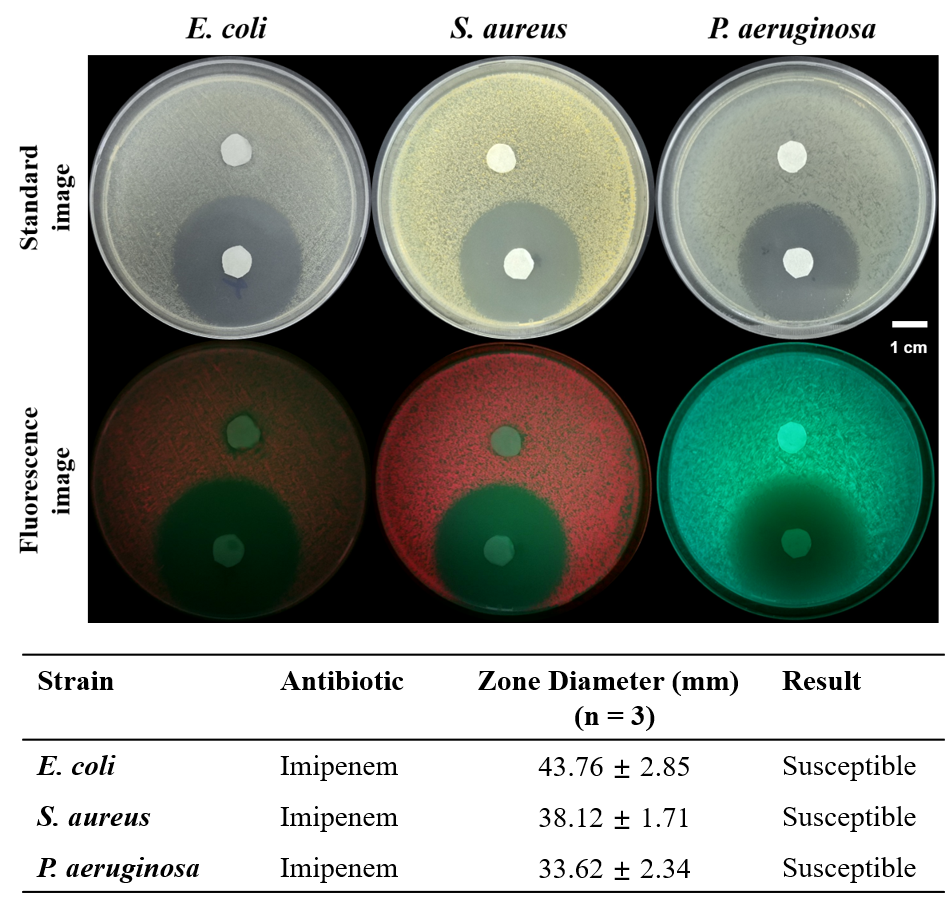


**Supplementary Figure 4.** Antimicrobial susceptibility of the bacterial strains to imipenem.

Antimicrobial susceptibility of *E. coli*, *S. aureus*, and *P. aeruginosa* to imipenem was assessed using the Kirby–Bauer disk diffusion method. Representative images of inhibition zones around imipenem disks are shown. Zone diameters were measured and susceptibility was interpreted according to CLSI criteria. Quantitative measurements and susceptibility classifications are provided in the table. Data are presented as mean ± SD.

1. [↑](#endnote-ref-1)
